# Organic Electronics—Microfluidics/Lab on a Chip Integration in Analytical Applications

**DOI:** 10.3390/s23208488

**Published:** 2023-10-16

**Authors:** Ruth Shinar, Joseph Shinar

**Affiliations:** 1 Electrical & Computer Engineering Department, Iowa State University, Ames, IA 50011, USA; 2 Physics & Astronomy Department and Ames National Laboratory—USDOE, Iowa State University, Ames, IA 50011, USA

**Keywords:** lab-on-a-chip, microfluidics, organic electronics, integrated sensors

## Abstract

Organic electronics (OE) technology has matured in displays and is advancing in solid-state lighting applications. Other promising and growing uses of this technology are in (bio)chemical sensing, imaging, in vitro cell monitoring, and other biomedical diagnostics that can benefit from low-cost, efficient small devices, including wearable designs that can be fabricated on glass or flexible plastic. OE devices such as organic LEDs, organic and hybrid perovskite-based photodetectors, and organic thin-film transistors, notably organic electrochemical transistors, are utilized in such sensing and (bio)medical applications. The integration of compact and sensitive OE devices with microfluidic channels and lab-on-a-chip (LOC) structures is very promising. This survey focuses on studies that utilize this integration for a variety of OE tools. It is not intended to encompass all studies in the area, but to present examples of the advances and the potential of such OE technology, with a focus on microfluidics/LOC integration for efficient wide-ranging sensing and biomedical applications.

## 1. Background

As is well known, the development of microfluidic or lab-on-a-chip (LOC) technology has enabled versatile processing of minute amounts of fluids (pico- to nanoliters) in miniaturized micro- and nano-size structures. The design of different microfluidic chips and the advantages of the technology in biomedical applications such as disease diagnosis, single-cell and single-molecule analyses, separation processes, chemical synthesis, and various sensing applications, including drugs, using low-cost, disposable device designs have been widely reported and reviewed [1,2,3]. In addition to current and expected advances, drawbacks of microfluidic devices and challenges have also been summarized [1,2,3].

Microfluidic devices are central to point-of-care (POC) diagnostics (see [2,3] and references therein). As such, the technology and its applications have been employed in conjunction with various systems, including integrating biosensors with a microfluidic chip for POC testing.

In a very recent review, Pal et al. [3] surveyed the recent advances in microfluidic drug sensing and screening. The survey included sensing techniques and materials, the morphology and structure of microfluidic channels, and applications of drug sensing. It also identified many companies that have commercialized such devices in which microfluidic chips have been integrated with electrochemical and optical detection elements in POC diagnosis, cell analysis, food safety, etc. Current challenges include, e.g., fabrication of valves in hard substrates such as glass and silicon, simplification of the manufacturing process, complete integration of the system in a single device, and the effects of nonspecific molecules. Pa et al. concluded that many drugs are suitable for microfluidic detection using, e.g., the optical properties of DNA and various metal nanoparticles. 

POC analytical tools are often optical or electrochemical and, as shown in the following sections, organic electronics (OE) is progressing in using such detection/monitoring tools with, e.g., organic light-emitting diodes (OLEDs), organic and perovskite photodetectors (OPDs and PPDs, respectively), and organic electrochemical transistors (OECTs) in optical and electrochemical (bio)chemical sensing and biomedical applications.

OE includes various thin-film devices, including polymers and small-molecule devices, that are based on π-conjugated semiconductors (namely, extended π-electron systems due to the presence of alternating single and double bonds). Such devices include OLEDs that are used successfully in display technology and are promising for solid-state lighting (SSL), optical analyte sensing, and various (bio)medical applications [4]. Other OE devices are organic and hybrid solar cells and related OPDs and PPDs, as well as organic-field-effect transistors (OFETs) [4]. The integration of OE devices with microfluidics and LOC is, therefore, promising for compact, potentially wearable efficient sensors, medical monitors, and therapeutic devices. As OE-related biomedical and sensing applications have been widely reported and reviewed [5,6,7], in this review, we do not elaborate on such applications but focus, instead, on examples of broad OE tools-microfluidic/LOC integration and its potential. However, our review is not intended to be inclusive. 

For clarity, this Background section continues with subsections that discuss the different OE devices—OLEDs, OPDs, PPDs, OLED–OPD combinations, and various OFETs. Then, in Section 2, our discussion of OE-microfluidics/LOC integration follows the same structure, starting with OLEDs, OPDs, and PPDs, and continuing with OFETs, including electrolyte-gated OFETs (EGOFETs) and organic electrochemical transistors (OECTs).

### 1.1. OLEDs

**Figure 1:**
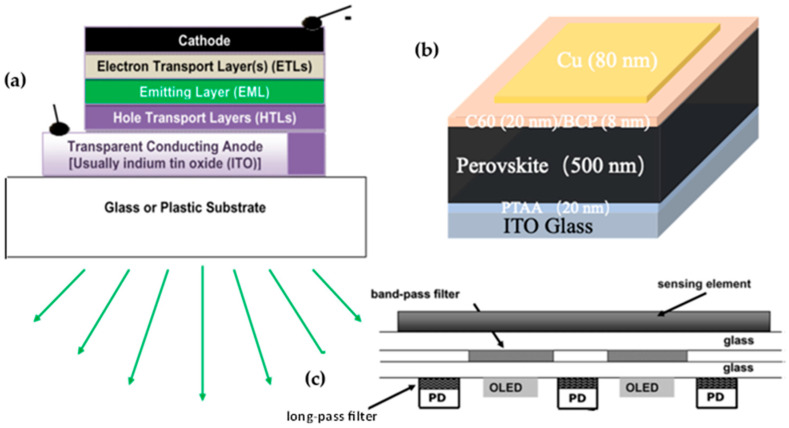
(**a**) Basic structure of an OLED. Reproduced from Shinar and Shinar [8] with permission from IOP Publishing. (**b**) The structure of a broad-band PPD; BCP and PTAA are bathocuproine and poly[bis(4-phenyl)(2,4,6-trimethylphenyl)amine, respectively. Reproduced from Wolanyk et al. [16] with permission from Elsevier B.V. (**c**) Schematic of the fully integrated OLED/sensing film/thin film PD array in a “back detection” configuration. Reproduced from Shinar and Shinar [8] with permission from IOP Publishing.

OLEDs are emitters that have the advantage of being flexible when fabricated on plastic substrates or on very thin glass (Figure 1a [8]). They are bright, have a wide viewing angle, are light in weight, and are thin (sub-micron thickness, excluding the substrate). OLEDs present superior attributes in comparison to liquid crystals in displays. They generally consist of several thin-film layers, including an emitting layer and charge transport layers, sandwiched between two electrodes, one of which is transparent. As OLEDs are fabricated on glass or plastic substrates, they are compatible with microfluidic channels. Efficient blue, green, red, and white OLEDs, including flexible OLEDs with a thickness typically < 0.4 µm per unit, excluding the substrate, have been commercialized for display and lighting applications. Typical OLED pixel sizes range from a few µm to ~1 cm^2^. Importantly, OLEDs have been employed in a variety of applications of optical sensing of (bio)chemical analytes, with the OLEDs serving as thin excitation sources [8,9,10,11,12,13,14,15]. In one brief example of operation, OLEDs, like every other light source, excite an analyte-sensitive dye that is embedded in a polymeric or other matrix. The dye’s photoluminescence (PL), monitored by a photodetector (PD) (see Figure 1b, an example of a broad-band perovskite-based PD), is affected by the analyte’s concentration, enabling its monitoring. Figure 1c shows a schematic of the fully integrated OLED/sensing film/thin film PD array in the “back detection” configuration [16]. 

OLEDs can also be used for monitoring analyte-induced changes in light absorption. They have also been utilized in medical applications, including photodynamic therapy [17,18], with OPDs [18,19,20,21,22,23,24,25], and in microfluidic structures (as shown below). A possible disadvantage of flexible OLEDs is their long-term stability, although disposable sensors are often desired.

Liquid OLEDs have also been developed [26,27]. These OLEDs utilize solvent-free emitting liquid organic semiconductors that, despite being generally inferior to solid-state OLEDs, can be more easily replaced when degraded and are less prone to, e.g., stretching and change in shape, displaying potential suitability for some applications. As described below, liquid OLED-microfluidic integration for on-chip designs have also been developed.

### 1.2. OPDs, PPDs, and OLED–OPD Combinations

Like OLEDs, OPDs and PPDs are π-conjugated organic thin layer structures that have also been successfully utilized in (bio)chemical analyte sensing and in medical applications [19,20,21,22,23,24,25,28,29]. OPDs and PPDs are thin-film devices of a solar cell structure (Figure 1b) that, depending on their active layer(s), absorb light at different wavelength ranges—narrow or broad [30,31,32,33].

**Figure 2:**
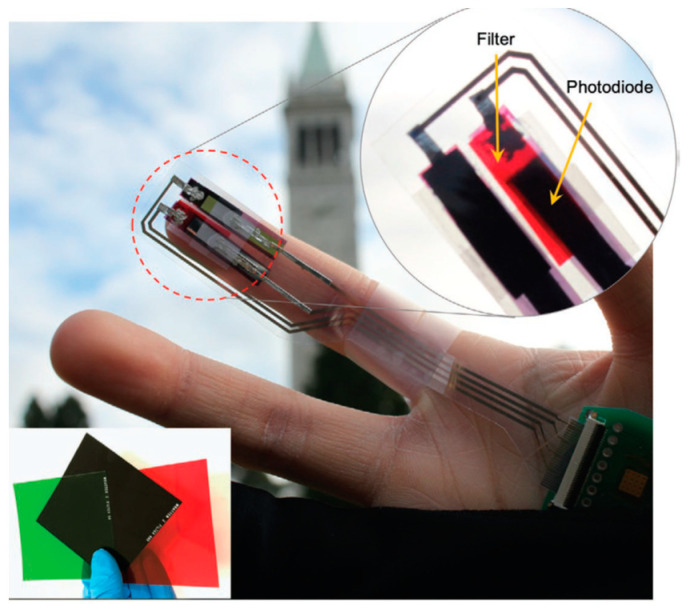
A photograph of the ambient light oximeter worn on the index finger, which is connected to a wireless circuit board. The oximeter is composed of two printed OPDs with spectral filters. Red, NIR, and green spectral filters are shown in the inset picture. Reproduced from Han et al. [34] with permission from Wiley–VCH GmbH.

Recent reviews have discussed theprogress in OPDs for, e.g., next-generation wearable devices and other biomedical uses (Figure 2 [34]). The integration of OPDs with other OE devices, such as OLEDs, has also been addressed [23,34,35,36]. 

Other examples of OLED and OPD combinations include flexible low-power oximeters [23,37] and other OLED–OPD-based photoplethysmography (PPG) sensors that are used for monitoring vitals, such as heart rate and blood oxygenation [23,38,39,40]. A PPG sensor operating in reflectance mode was demonstrated, using an OPD that was fabricated monolithically with an OLED on a glass substrate [38]. Recently, the development of a flexible PPG sensor with interfering light-and-motion compensation, with the aid of an additional reference OPD channel, was demonstrated [40]. Another example is optical muscle contraction sensors that measure tissue oxygenation via measuring blood pressure cuff-induced ischemia [19]. 

**Figure 3:**
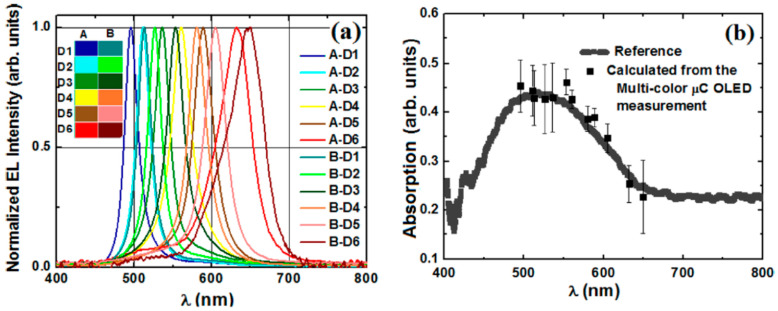
Toward a spectrometer-on-a-chip: (**a**) EL spectra of a 2D combinatorial array of microcavity (µC) OLEDs. (**b**) Absorption spectrum of a P3HT film on glass, measured with the µC OLED array and a PMT vs. the reference using a tungsten lamp. Reproduced from Liu et al. [41] with permission from AIP Publishing.

An OLEDs–OPDs combination of advantageous sensitive-compact-device designs enabled light-absorption measurements (e.g., a spectrometer-on-a-chip, as shown in Figure 3) [15,41]. The compact spectrometers-on-a-chip were of wavelengths that ranged from near UV to red [4,15,41,42]. They were used to monitor the absorption spectra of thin layers, such as Alexa fluor that is of use in immunoassays. 

In sensing applications, OPDs integrated with OLEDs (schematically shown in Figure 1c) were shown to enable the sensitive detection of different analytes, such as gas-phase and dissolved oxygen, glucose, lactate, ethanol, anthrax lethal factor, and hydrazine, in small-size monitors [4,8,9].

In another recent development, we successfully used PPDs to optically monitor oxygen and glucose [16]. PPDs are generally more sensitive than OPDs; they can detect very low-intensity light signals (<1 pW/cm^2^ [30]) and, importantly, respond very quickly, depending on their size, to changes in light intensities [32]. Perovskite-based solar cells and, thus, PPDs are of continued interest, due to their excellent optoelectronic properties [43,44]. The potential of nanocrystalline hybrid perovskite thin films to quickly and efficiently detect explosives’ vapor by film PL quenching was also demonstrated recently [45]. In particular, the authors of that study showed that by tuning the perovskite nanocrystals to low dimensionality and a high surface area, they could dramatically enhance the quenching of the films’ photoluminescence (PL) by dinitrotoluene (DNT)—up to 70% quenching by 3 min exposure to ppb levels; the PL recovered by 50% in 1 min. The long-term stability of OPDs and PPDs presents a challenge, unless the sensors are disposable. 

### 1.3. OFETs

In addition to the optical devices described above, OFETs have drawn strong attention as flexible versatile disposable biosensors for, e.g., POC applications [46]. In brief, OFETs comprise interfacing organic semiconductor and dielectric layers and gate (*G*), source (*S*), and drain (*D*) electrodes. Various OFET structures, depending on the layers’ order and the relative location of the conducting channel that connects the source to the drain, have been studied. Device operation depends on the voltages applied to the gate (*V_GS_*) and the drain (*V_DS_*) relative to a grounded source electrode. OFET performance is described by, e.g., the drain current *I_D_* vs. *V_GS_* at a fixed *V_D_*_S_ (transfer characteristic) and by the threshold voltage (*V_th_*).

Among OFETs, electrolyte-gated transistors (EGTs) for biosensing and other related applications, showing potential for POC testing, have been widely developed and reviewed [47,48,49]. Organic semiconductor-based EGTs are divided into two main classes, electrolyte-gated organic field-effect transistors (EGOFETs) and OECTs [47]. Torricelli et al. [47] described EGTs in detail, including materials, fabrication, structures, and operation. In a basic EGT structure, the gate and the semiconducting channel interface with the electrolyte. The magnitude and polarity of *V_GS_* result in ion drift from the electrolyte to the channel, affecting its electronic charges. This ionic–electronic modulation, which is due to the accumulation or depletion of ions, results in variations in the channel conductivity.

In the case of EGOFETs, which operate at low biasing, the channel is ion-impermeable; the ionic–electronic interaction occurs only at the electrolyte–channel interface. Upon biasing the gate and drain (with the source grounded) an electrical double layer is formed at the electrolyte–channel interface. A major advantage of EGOFETs is their much higher gate capacitance—up to ~1000 higher—than that of non-electrolytic OFETs [50]. Consequently, the formers’ biasing voltages are typically < 1 V, versus > 10 V or even higher for the latter. The aqueous electrolyte and low potential render EGOFETs ideal for the detection and quantification of biological molecules inside aqueous media.

Doumbia et al. [51] recently developed microfluidic-integrated EGOFET sensors for rapid in situ label-free DNA hybridization monitoring, highlighting their potential for biological applications (see Section 2.2.1). These organic bioelectronic technologies advanced rapidly, with recent demonstrations of single-molecule detection with EGOFETs [52].

In OECTs, the channel is ion-permeable, as is the case for the organic mixed ionic–electronic conductor poly(3,4-ethylenedioxy thiophene):polystyrene sulfonate (PEDOT:PSS). The OECT device operation is based on the channel’s electrochemical doping/de-doping, with ions entering the active region following gate biasing. Other leading OECT polymers, listed by Torricelli et al. [47], include poly [3-(5-carboxypentyl)thiophene-2,5-diyl] (P3CPT), poly(2-(3,3′-bis(2-(2-(2-methoxyethoxy)ethoxy)ethoxy)-[2,2′-bithiophen]-5-yl)thieno [3,2-*b*]thiophene) (p(g2T-TT)), poly [2,6-(4,4-bis-potassium butanylsulfonate-4H-cyclopenta-[2,1-*b*;3,4-*b*′]-dithiophene)-*alt*-4,7-(2,1,3-benzothiadiazole)] (PCPDTBT-SO3K (also known as CPE-K), poly(benzimidazo-benzo-phenanthroline) (BBL), and poly[1,2,3, 6,7,8-hexahydro-1,3,6,8-tetraoxo-2,7-bis(7-oxo-3,6,9,12,15-pentaoxahexadec-1-yl)benzo[lmn][3,8]phenanthroline-4,9-diyl][2,2′-bithiophene]-5,5′-diyl](p(gNDI-T2)).

**Figure 4:**
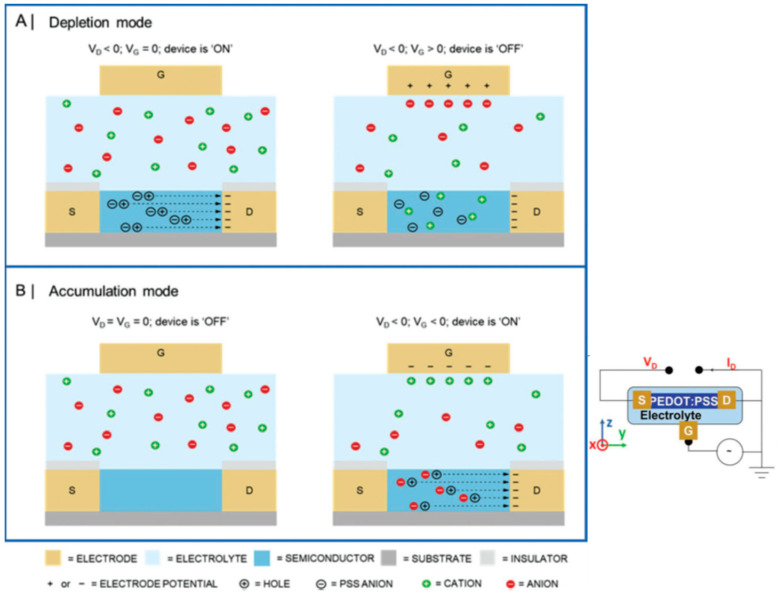
Left: operating principle of (**A**) depletion-mode and (**B**) accumulation-mode organic electrochemical transistor, highlighting the formation of an electrical double layer across the bulk of the channel. Reproduced from Marks et al. [56] with permission from Wiley-VCH GmbH. Right: schematic of the structure of an OECT. Reproduced from Wang et al. [57] with permission from Wiley-VCH GmbH.

OECTs are electronic devices that utilize organic materials to efficiently transduce ionic signals into electronic signals, making them valuable tools for various applications, particularly in biosensing and biomedical fields. The OECT structure and operating principle (Figure 4), as well as some specific applications, are described below [6,53,54,55,56]. The mixed ionic–electronic PEDOT:PSS conductor with its electron-rich polythiophene backbone is p-type doped, operating in depletion mode. 

Figure 4 shows the operating principle of OECTs. The channel is in contact with an electrolyte, in which the gate electrode is immersed. Under suitable voltages of the gate and the drain, ions in the electrolyte, which are aqueous when dealing with biological systems, electrochemically (de)dope the organic channel, changing its conductivity; the drain current then probes the doping state (redox state) of the channel. The following paragraphs present some detection examples.

There are numerous examples of OECTs in biosensing and related applications, including immunosensors, pathogen sensing, and neuromorphic sensing. OECTs can be functionalized with biomolecules (e.g., pathogens, antibodies, enzymes, proteins, or DNA probes) that selectively bind to target analytes. When the target analyte interacts with the functionalized surface, it leads to changes in the channel ionic concentration, which is transduced into an electronic signal. OECTs can be used also for monitoring the effects of drugs on cells or tissues in real time.

In a specific example, OECTs were successfully used to record in vivo neural activities [58,59]. Recently, nanobody-functionalized, high-gain circuitry OEC structures enabled fast (<15 min) quantification and sensitive detection, down to a single molecule of specific protein antigens (e.g., green fluorescent protein, COVID-19), including detection in small-volume unprocessed human saliva and serum [60].

OECT-based wearable and implantable devices have also been reported. These devices have the potential to revolutionize healthcare by providing real-time, accurate, and non-invasive monitoring of various physiological parameters. They can enhance patient care, enable early disease detection, and contribute to developing personalized medicine approaches.

For example, a textile wearable glucose OECT sensor has been developed [61], as well as a textile wearable sensor, for both Cl^-^ ions and pH [62].

**Figure 5:**
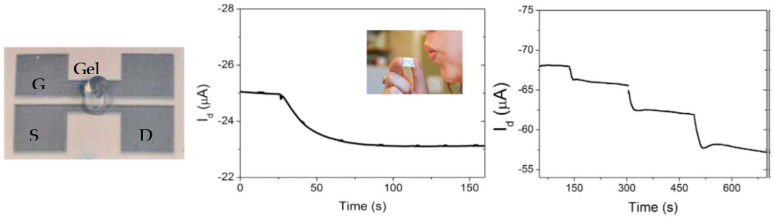
Concept of the alcohol OECT-breathalyzer. Left: the OECT is printed on paper and comprises a PEDOT:PSS channel and source (S), drain (D), and gate (G) electrodes. The enzyme electrolyte gel is deposited on the OECT, bridging the channel and the gate. Center: drain current I_d_ response of the OECT upon exposure to ethanol. Right: step change in I_d_ when adding 0.0004%, 0.004%, and 0.04% ethanol solutions. Reproduced from Bihar et al. [63] with permission from Springer Nature.

Bihar et al. [63] demonstrated a disposable OECT enzymatic alcohol breathalyzer, printed on paper and utilizing an electrolyte gel. Upon exposure of the OECT to alcohol, the drain current decreased, enabling sensitive detection (see Figure 5).

**Figure 6:**
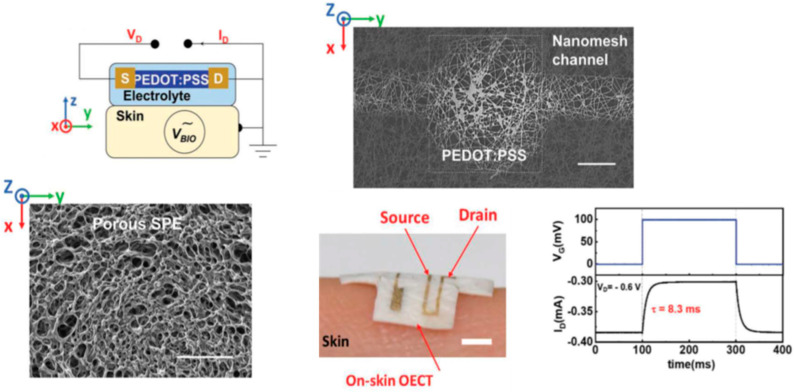
Top: schematic showing how the OECT works as an on-skin electrode, in which human skin is used as the gate of the OECT (left); SEM image of the channel part of the OECT, scale bar 200 μm (right). Bottom: SEM image of the porous SPE, scale bar: 200 μm (left); a photo of the on-skin OECT, scale bar 2 mm (center); the response speed of the on-skin OECT (right). Reproduced from Wang et al. [57] with permission from Wiley-VCH GmbH.

Wang et al. [57] recently described a gas-permeable, porous, solid-state polymer electrolyte (SPE) embedded in a nanomesh OECT as an on-skin active electrode for fast electrocardiogram (ECG) signal acquisition. Figure 6 shows the operation and attributes of the on-skin OECT.

In a recent study, an OECT was integrated with a PPD on a flexible substrate, coupling the excellent optoelectronic performance of the latter with the high transconductance (*g*_m_ = *∂I*_DS_/*∂V*_GS_) of the OECT [64]. The device demonstrated a fast response and sensitive tracking of PPG signals from different positions in the human body.

The examples presented in this Background section already allude to the potential of integrating the OE devices with microfluidics, as detailed below.

The advantages of OFETs and OECTs in sensing and biomedical applications are clearly shown in the above-mentioned versatile examples. However, OFETs can present a slow response time that is limited by ion migration into or out of the channel. Microfabricated devices show faster response times. Niu et al. [65] listed additional disadvantages of OFETs and OECTs, including the need to improve sensitivity, selectivity, reproducibility, and stability. As discussed below, an exciting new direction involves the seamless integration of compact and highly sensitive OE devices with microfluidic channels and lab-on-a-chip structures. This integration and development of new materials and device structures should address these potential limitations.

## 2. OE-Microfluidics/LOC Integration

As shown in this section, OE-microfluidic/LOC integration has already resulted in promising devices for a variety of applications. Improving some of the OE devices’ attributes (e.g., design, long-term stability, response time) and the utilization of flexible substrates are expected to further improve this integration and its technological appeal.

### 2.1. Optical Applications

In optical applications, the first step was combining OLEDs or OPDs with microfluidic designs. The combination of OLEDs–OPDs-microfluidics followed. Some examples of early OLED- and OPD-microfluidic channels integration for, e.g., sensing and imaging, are briefly described next.

#### 2.1.1. OLEDs-Microfluidic Integration

On-chip multianalyte sensing with OLEDs as the excitation source has been studied in both absorption and photoluminescence (PL) modes [6]. Camou et al. [66] presented a step toward a lab-on-a-chip device by integrating microchannels in polydimethylsiloxane (PDMS) with a green OLED used to excite the fluorescence of the dye Rhodamine B, which is used as a label of biomaterials in, e.g., protein optical detection. The potential and the drawbacks of such a device, many of which have been addressed already, were discussed. Similarly, 520 nm green polymer LED (PLED) excitation was used in conjunction with lab-on-a-microchip for dye fluorescence analysis that was detected by a spectrometer. The authors concluded that the OLEDs-microfluidics integration is expected to be beneficial for POC fluorescence assays [67].

Different applications and approaches have also been reported. Edel et al. [68] described the integration of a fluorescent PLED that was applied for the detection of fluorescein and 5-carboxyfluorescein with a microfabricated microscale capillary electrophoresis (CE) device. Yao et al. [69] developed an imaging-detection system using an OLED as a 2D light source for on-chip isoelectric focusing of fluorescent proteins. Optically pumped organic semiconductor lasers were also integrated with LOC on plastic for, e.g., monitoring labeled antibodies [70].

**Figure 7:**
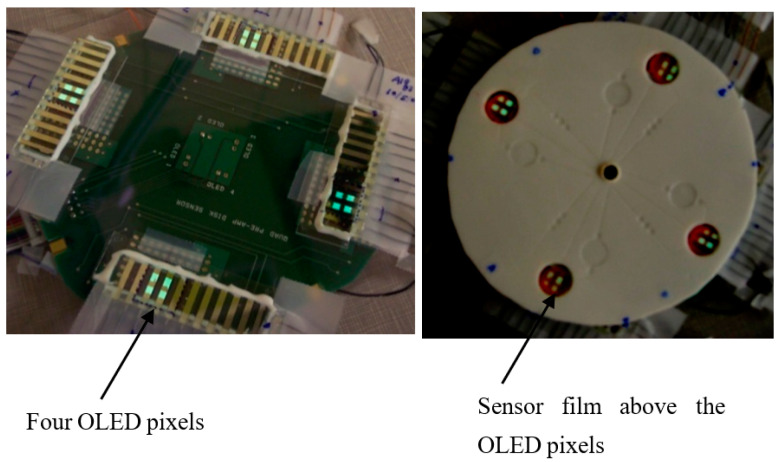
Images of the CD–OLED–PD sensing platform. Left—the PD array and the green OLED pixels in operation; right—the complete device with the sensor films (pink) on the PP CD; the lit OLED pixels are also visible. Reproduced from Vengasandra et al. [12] with permission from RSC Publishing.

Shinar et al. [12] fabricated sub-micron thick OLED pixels and successfully integrated them with LOC (Figure 7). Specifically, a lab on a compact disk (CD) was fabricated. Microfluidic features were generated in the CD, which was made up of foamed polypropylene (PP), by ultrasonic micro-embossing. The microfluidic features included channels, reservoirs, valves, and reaction chambers, enabling, together with compact OLEDs and 2 mm thick Si PDs, the simultaneous monitoring of glucose, lactate, ethanol, and dissolved oxygen (DO) in four separate single CD segments, using a standard PC–CD player. Glucose, lactate, and ethanol were monitored following their oxidation in sealed cells in the presence of oxygen and their specific oxidase enzyme. The stoichiometric reactions resulted in DO consumption, which was monitored via the PL decay time of an oxygen-sensitive dye (Pt octaethylporphyrin, PtOEP, embedded in a polystyrene film and serving as the bottom of the reaction chamber) following an OLED excitation pulse. This monitoring followed controlled CD rotation, which enabled specific fluid flow, due to an appropriate burst valve, for reagent mixing (e.g., test solution and oxidase enzyme). The limit of detection (LOD) was ~0.02 mM for each of the bioanalytes. Overall, the results demonstrated sensitive and accurate simultaneous monitoring of multiple analytes (including real-world samples), using the compact OLED-Bio CD–PD platform, and its potential field-deployability.

Marcello et al. [71] presented a successful integration of deep blue OLED excitation source with a biochip for detection of nanograms of antigen, utilizing commercially available antibody conjugated to Alexa Fluor, to which the OLEDs were coupled. This work, like the previous study, demonstrated the potential for developing such integrated portable devices for POC testing.

**Figure 8:**
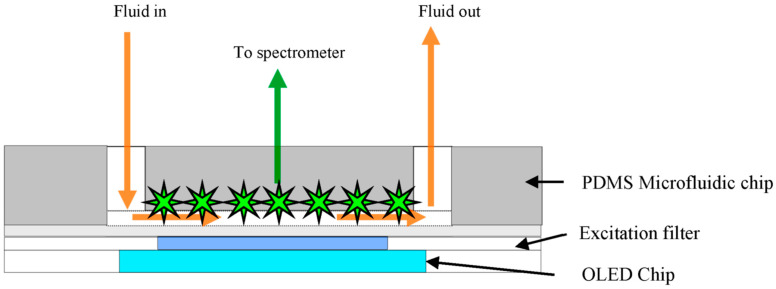
OLED source-filter-chip-detector setup. Reproduced from Acharya et al. [72] with permission from MDPI.

Acharya et al. [72] integrated a PDMS microfluidic structure, prepared by soft lithography, and OLEDs. The detection of anti-sheep IgG labeled with Alexa fluor 488 and sandwiched between two antibodies was based on fluorescence, with the results showing promise for fast protein detection. Figure 8 shows the design of the integrated OLED-microfluidic chip that is suitable for compact immunoassays.

A recent review summarized advances in solid-state OLEDs and their combination with microfluidic channels for biomedical applications [6]. Advances were possible due to significant improvements in OLEDs’ brightness, stability, and external quantum efficiency (EQE), as well as their compatibility with different substrates (including flexible plastic) and their promise as wearable devices [73,74,75]. The review by Murawski and Gather [6] also addressed some strategies for OLED miniaturizing and patterning, which are ongoing efforts for broadening their use in biomedical applications.

**Figure 9:**
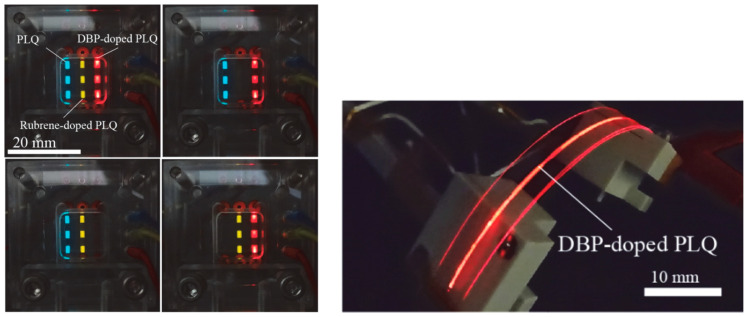
Left: multi-color EL emissions from the microfluidic OLED with 1-pyrenebutyric acid 2-ethylhexyl ester (PLQ), rubrene-doped PLQ, and DBP-doped PLQ. The target light-emitting pixels are simply controlled by passive matrix addressing. Right: EL from the microfluidic OLED ribbons. Reproduced from Kasahara et al. [80] with permission from J. Photopolym. Sci. Technol.

The OLEDs described so far were solid-state devices. While still requiring further optimization, on-demand flexible multi-color liquid organic semiconductor (LOS) emitters were generated by injecting the emitting materials into single µm-thick microchannels sandwiched between the electrodes and LOS-microfluidics integration for on-chip designs were demonstrated [76,77,78,79,80]. Initially, the devices were not used for any application, but the fabricated multi-color emitters and flexible devices presented the capability of the integration for potential future sensing and display applications (see Figure 9).

Standard solid-state OLEDs, as well as LOS-based OLEDs, typically present broad-band emission profiles, which can lead to interference in analyte detection. Microcavity OLEDs provide a solution to this challenge [13,15,41], but other approaches were also developed for narrowing the light-source-emission profile. A recent review described such advances in light-source-microfluidic integration [80].

**Figure 10:**
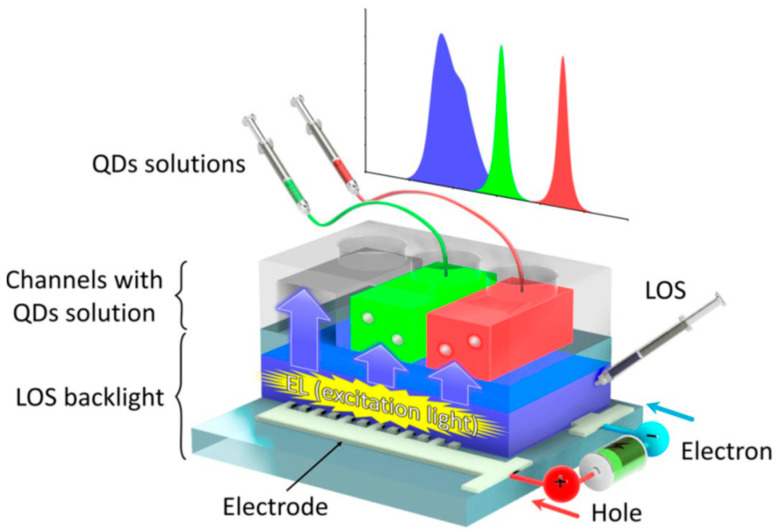
Concept of the liquid/solution-based microfluidic QLEDs. Proposed microfluidic QLEDs consist of a LOS backlight and a QDs-solutions layer. The channels for the QDs solutions are stacked on the LOS backlight. The QDs solutions work as color converters and color filters to emit high-color-purity lights with narrow FWHMs. Reproduced from Kawamura et al. [81] with permission from Springer Nature.

Kawamura et al. [81] developed all-liquid quantum dot (QD) LEDs (QLEDs). These devices contain blue LOS for backlight and narrow red (25.0 nm FWHM) and green (26.2 nm FWHM) emitting QD-solutions down-conversion filters (see Figure 10).

The QD water solutions excited by the LOS were in PDMS channels [81]. The authors suggested that the devices are promising for the next-generation flexible display; they are also potentially usable as light sources for bioanalytical and related applications.

Continued advances in OLED display may further enhance the use of OLEDs in medical and related applications. Recently, Muramatsu et al. [82] reported the development of a microfluidic multicolor display, based on mixing four primary colors of patterned OLED pixels. Among other potential applications, these may be promising as next-generation wearable displays. With wearable OLEDs already reported for medical applications [17,19,24,73], light sources with a small number of displayed colors may also be useful.

Further advances associated with OLED–LOC integration typically include the added integration with OPDs, as described below.

#### 2.1.2. OPDs-Microfluidic Integration

OPDs, like OLEDs, are thin-film devices based on π conjugated organic small molecule or polymers. They are basically solar cells operated at zero or low-negative voltage. The most efficient OPDs are typically the bulk heterojunction devices with a light absorbing donor:acceptor pair as the active layer; the donor:acceptor blend structure enables efficient electron–hole separation [83,84]. A common pair is the blend of P3HT:PCBM (poly(3-hexylthyophene):[6,6]-phenyl C61-butyric acid methylester), but other more stable and efficient non-fullerene donors, with efficiencies exceeding 17%, have been successfully used [83,84].

Similarly, PPDs are thin-film devices that are typically more efficient than OPDs. The hybrid perovskite devices present strong light absorption and better charge separation. Research for improving organic and hybrid solar cells and PDs is ongoing.

**Figure 11:**
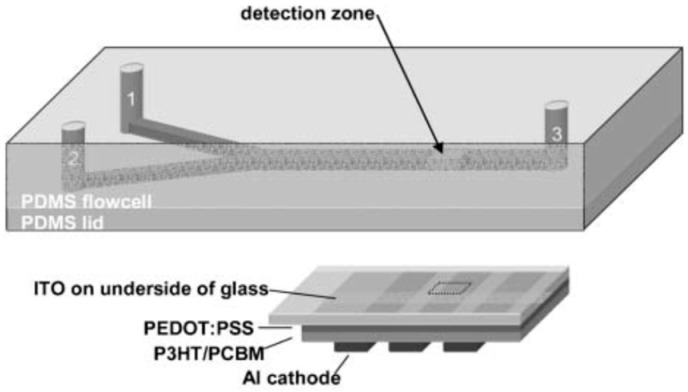
Microchip layout with inlets for premixed CPPO, dye, catalyst (1), and hydrogen peroxide (2), mixing channel, and outlet (3). The inlets are 400 mm wide, 800 mm deep and 1 cm long, while the mixing channel is 800 mm wide, 800 mm deep and 5.2 cm long. The active area of the photodiode used for CL detection is 1 mm × 1 mm. The photodiode is located at a position 1 cm downstream from the point-of-confluence of the two inlet streams. Reproduced from Wang et al. [86] with permission from RSC Publishing.

In initial studies, Hoffman et al. [85] described small-molecule OPDs as optical detectors for microscale chemiluminescent (CL) reaction assays. Later, Wang et al. [86] improved that assay using solution processed polymeric OPDs that previously were used for hydrogen peroxide monitoring via detection of the emission signal from a peroxyoxalate CL (PO-CL)-based assay. The active reaction ingredients were bis(2-carbopentyloxy-3,5,6-trichlorophenyl) oxalate (CPPO), the emitting dye 9,10-bis(phenylethynyl)anthracene (the emission spectrum of which overlaps the OPD’s spectral response), and hydrogen peroxide; 4-dimethylaminopyridine (DMAP) was added as a reaction catalyst. The authors showed that, with further improvement, such microscale OPDs with PDMS microfluidic chips can be useful for biochemical analyses and disease diagnostics, as they are sensitive, small in size, and, potentially, low cost (see Figure 11). In a further development of this approach, the authors demonstrated its application to antioxidant capacity screening [87].

Wojciechowsk et al. [88] developed a miniaturized CL immunoassay with disposable parts; small-size biosensors are essential for field deployability and POC testing. The biosensor integrated an OPD and a biochip; it detected Staphylococcal enterotoxin B at levels as low as 0.5 ng/mL.

A multiplexed pathogen CL biosensing platform integrating microfluidic channels in poly(methylmethacrylate) (PMMA) with an OPD array was also demonstrated [89]. Parallel analysis of the three pathogens *Escherichia coli* (LOD 5 × 10^5^ cells/mL), *Campylobacter jejuni* (LOD 1 × 10^5^ cells/mL), and *adenovirus* (LOD 1 × 10^−8^ mg/mL), including in a single sample, was performed successfully, demonstrating the ability of the integrated sensor to detect multiple bacteria and viruses simultaneously in future miniaturized devices.

A miniaturized cell-on-a chip, label-free analysis platform to monitor dynamic cell behavior was developed by Charwat et al. [90]. Fast (within minutes) information on cell numbers and intracellular granularity was obtained by light scattering from adherent cells and, when impedance spectroscopy was used, cell adhesion and cells interaction were simultaneously monitored.

Optical sensing chips that monitor both luminescence and absorption using OPDs fabricated on glass or a polymeric foil were also developed [91]. The sensor was used for monitoring gas-phase oxygen, carbon dioxide, and relative humidity via luminescence. Oxygen and carbon dioxide monitoring was also obtained via absorbance, and oxygen and pH could be measured in solution.

As shown below, OLED-OPD/LOC integration, in which two different OE devices are integrated with microfluidic channels, was also demonstrated successfully.

#### 2.1.3. Organic Electronic Light Source-OPD/LOC Integration

In the PL-sensitive analyte detection scheme, an excitation source excites a light-emitting molecule, the PL intensity and decay time (following pulse excitation) of which are analyte-dose dependent; a PD monitors this PL. For successful OLED–OPD or OLED–PPD combined operation, narrow OLED emission bands and suitable OPD or PPD absorption bands are required to enable sensitive detection with no interfering light from, e.g., the typically broad OLED’s EL spectrum that can reach the OPD. Recent advances in efficient multi-color narrow OLED bands, as well as broad and narrow band OPDs and PPDs [4,15,16,30,32,33,41,42], are very promising for achieving high sensitivity and low LOD without the need for external optical filters that can increase the monitors’ size, adversely affecting their field deployability.

**Figure 12:**
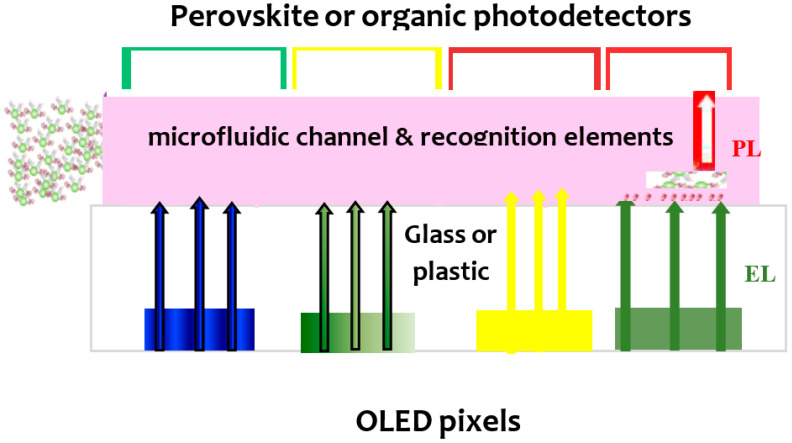
Schematics of a possible analyte monitoring operation mode of OLED–OPD or OLED–PPD integrated with a microfluid structure.

Figure 12 depicts the schematics of one operation mode of OLED–OPD or OLED–PPD integrated with a microfluidic channel, where analyte recognition elements are immobilized at the bottom of the microchannel. The analyte molecules introduced into the channel can be labeled with a suitable dye. Various-colored OLED pixels can be used with either broadband or narrow-band PDs.

An early review by Williams et al. [5] pointed to the potential of blending microfluidic/LOC with OE OLED and OPD technologies for PL-based analyte detection. The authors provided many examples and addressed the potential of monitoring multiplexed immunoassays using multiple OLED excitation wavelengths and suitable (tunable) OPDs. Therefore, only a few distinct highlights of early reports of different natures will be provided here.

There have been significant recent advances for this promising approach [92,93], including potential future integration with the highly sensitive PPDs that were recently reported for sensitive (bio)chemical analyte detection [16].

As mentioned, OE devices are easily and inexpensively fabricated at ambient temperatures and, thus, they are compatible with, e.g., PDMS microfluidic channels. Their fabrication via thermal evaporation of the various layers or solution processing and the principle of operation have been widely reviewed. Many high-throughput approaches of fabricating such devices are well established.

Early on, Pais et al. [94] described a disposable microfluidic lab-on-a-chip device with an integrated green OLED excitation source and OPD for sensitive fluorescence dye detection. The LOD was 100 nM for Rhodamine 6G and 10 µM for fluorescein. The detection of multiple dyes with the same device was also possible. The results suggested that such a portable device can be suitable for POC fluorescence testing. Indeed, such integration has since advanced.

**Figure 13:**
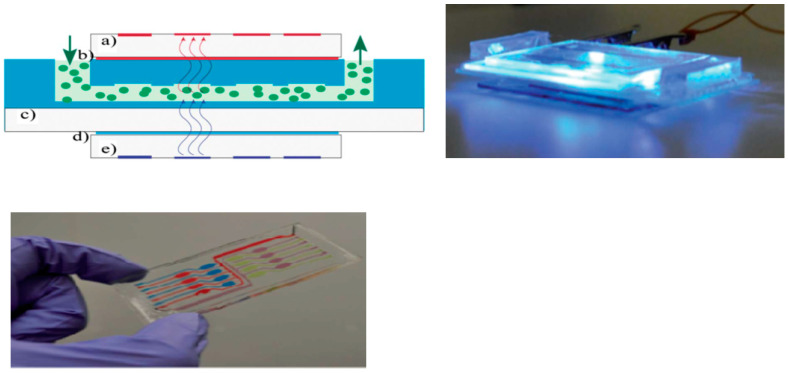
Top left: edge view of the schematic of the proposed fluorescence sensor: the microfluidic chip (**c**) is sandwiched between the OLED (**e**) and the OPD (**a**). The emission filter (**d**) is between the microfluidic chip and the OLED, while the excitation filter (**b**) is between the microfluidic chip and the OPD. Top right: fluorescence sensor with illuminated OLED. Bottom: microfluidic chip with 16 chambers (color dyes show the micro channels). Reproduced from Lefevre et al. [95] with permission from RSC Publishing.

Lefevre et al. [95] reported the first miniaturized fluorescent sensor utilizing a blue-emitting OLED and an OPD integrated with a PDMS microfluidic chip. The sensor successfully monitored the effect of a pollutant, an agricultural herbicide toxin, on green algal chlorophyll fluorescence. Figure 13 shows a schematic edge view and pictures of the sensor and the microfluidic chip. Suitable optical filters, placed in front of the OLED and the OPD, were developed to avoid interfering light, which adversely affects the LOD. This resulted in a high-sensitivity device, with a Diuron LOD of 7.5 nM.

Exner et al. [96] stacked three devices: an OLED, a1D photonic crystal (PC) as a tunable filter, and an OPD. This miniaturized optical sensor is suitable for a microfluidic setup. It can detect changes in the transmission of the PC; such changes can be generated by analytes in solution and in the gas phase. The sensor detected 10 ppm of ethanol vapor in nitrogen.

A selective, narrow emission OLED (peak emission 546 nm, 9 nm FWHM)/OPD (suitable for monitoring fluorescence at 586 nm) compact fluorescent sensor integrated with a PDMS microchip was used in a competitive enzyme-linked immunosorbent assay (ELISA) for alkylphenol polyethoxylate (APE), the breakdown products of which included toxic chemicals. In one approach, the anti-APE antibody was immobilized on the surface of the PDMS channel [97].

Following the immobilization of the antibody in the microchannel, a mixture of APE and horseradish peroxidase (HRP)-labeled APE was added. Next, Amplex Red reagent and H_2_O_2_ continuously flowed into the channel at different flow rates. The 1:1 stoichiometric oxidation reaction of the latter in the presence of HRP produced Resorufin, a fluorescent probe, the concentration of which and that of the APE could then be calculated; the LOD was ~1 ppb.

Shu et al. [98] reported the first low-cost optical sensor based on a solution-processed organic-electronics-excitation source, i.e., a blue organic light emitting an electrochemical cell (OLEC) and a P3HT:PCBM OPD that were mounted on a microfluidic glass chip, together with two orthogonal linear polarizers that were used as filters. The latter enabled monitoring dyes with close excitation and emission peaks, such as fluorescein amidite (FAM), the PL excitation and emission peaks of which were at 492 and 518 nm, respectively. Both the OLEC and the OPD had the same inverted layer structure of the ITO cathode/ZnO/active layer/PEDOT:PSS anode. The OLEC active layer was a blue-emitting polymer, a solid electrolyte, and a dopant. The PN-junction within the active layer of the OLEC was achieved via electrochemical doping and ion redistribution.

**Figure 14:**
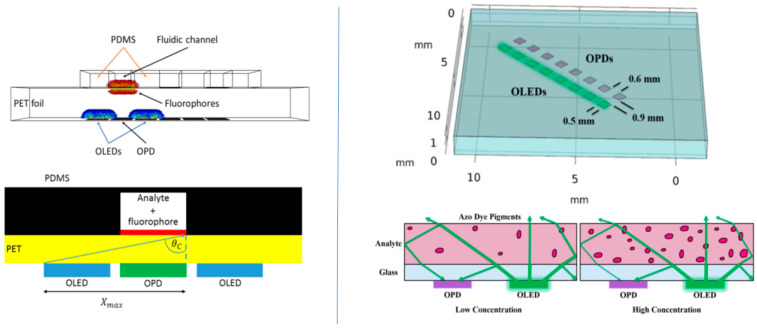
Left panel: model for ray-tracing simulations; (top) matrix of OLEDs emitting into the substrate and OPDs on PET foil. Fluorescence spots are above OPDs on the other side of the PET foil. A PDMS microfluidic chip is placed such that the fluidic channels are above the OPDs. The PDMS fluidic channels are placed above the fluorophore arrays; (bottom) 2D view of system with black PDMS microfluidics to prevent stray light. Reproduced from Titov et al. [92] with permission from IEEE Sensors Journal. Right panel: (top) geometrical model of the OLED–OPD unit layout with 8 units on one test chip; (bottom) schematic of the azo dye concentration-dependent absorbance of OLED light inside the analyte; (bottom left) the light propagated partly inside the glass substrate and was partly reflected back onto the OPD; (bottom right) for higher concentrations of nitrite standard sample and consequently higher concentrations of the azo dye, the light showed increased absorption inside the chamber at the azo dye pigments. Thus, only a fraction of the initial amount of light was reflected back onto the OPD. Consequently, the photocurrent decreased. Reproduced from Titov et al. [93] with permission from MDPI.

More recently, Titov et al. [92] described multiplex optical sensors that were fabricated by integrating 18 blue OLEDs and 18 OPDs on flexible polyethylene terephthalate (PET) attached to a microfluidic system. The OLEDs and the OPDs were fabricated on the same side of the PET foil (i.e., a “back detection geometry”) with the analyte and fluorophore located in the PDMS channel above the OPDs (see Figure 14). This structure, however, required improvements in the design and in the device size (due, e.g., to leakage currents), the use of suitable optical filters, and OLEDs and/or OPDs with narrow EL and absorption bands, respectively. The authors concluded that the absorption signal dominated their system and that, consequently, narrow-band OPDs were preferable. Alternatively, they suggested smaller devices (e.g., 1 × 1 mm^2^) to reduce stray light and reduce the overall device size.

A 12.5 × 12.5 mm^2^ 8 OLED–OPD pair array on a common glass substrate with a reflection-type design was later developed for monitoring nitrites in solution (see Figure 14) [93], e.g., for toxicity testing of drinking water or aquaculture systems. Such a structure is suitable for integration with a microfluidic structure. The nitrite concentration determination was based on the Griess diazotization reaction and the absorption of the azo dye product, whose concentration depends on that of the nitrite; the LOD was 46 µg/L (1.0 µM). The OLED peak emission was at ~512 nm and the maximal sensitivity of the OPD was at ~530 nm, so that the OPD measured changes in the OLED’s emission that propagated in the glass substrate, due to total internal reflection (TIR). As the concentration of the azo dye increased, the photocurrent measured by the OPD decreased, due to both the change in the solution refractive index and the light absorbance by the dye. That is, the critical angle for TIR in the glass and the light absorption increased as the analyte concentration increased, so that the guided light in the glass, due to TIR, which reached the OPD, decreased.

OFET-microfluidics integration was reported more extensively than optical OE integration with microfluidics. Significant advances in integrating various OFET structures with microfluidics for various applications were widely reported; some recent examples are provided below.

### 2.2. OFET—Microfluidics Integration

Various integrated microfluidic OFET devices in different bioelectronic applications have been widely reported and reviewed; some highlights are presented here [48,50,99,100,101]. A recent review, which briefly discussed FETs, described advances in the integration of various FETs with microfluidic structures and their potential in (bio)chemical applications [101].

**Figure 15:**
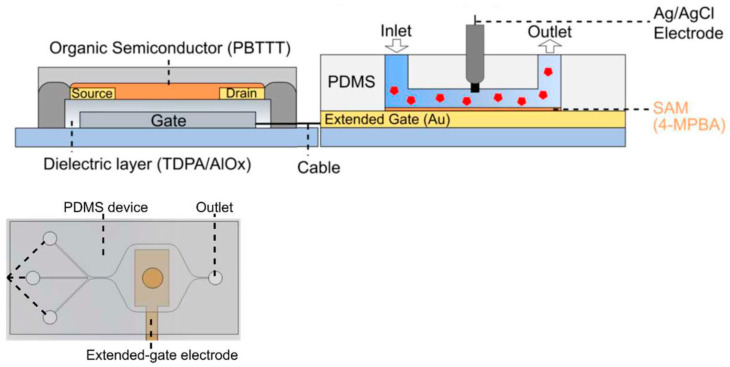
Top: schematic illustration of the fabricated extended-gate OFET sensor integrated with the microfluidic system. The extended-gate electrode was functionalized with 4-MPBA. Bottom: schematic top view of the microfluidic system combined with the extended-gate type OFET. Reproduced from Didier et al. [102] with permission from Wiley–VCH GmbH.

An extended-gate OFET (with the extended gate—that is, the sensing electrode—separated from the OFET drive component) integrated with a microfluidic was used successfully for monitoring glucose in real time [102]. Reversibly monitoring pseudo-consumption and the release of glucose in liver cells was also demonstrated. Detection was achieved by functionalizing the Au-extended gate with 4-mercaptophenylboronic acid (4-MPBA) that recognized glucose, forming an anionic boronate ester that resulted in a change in the OFET’s drain current *I_DS_* and *V_th_*. Figure 15 shows the schematic of the extended-gate device integrated with the microfluidic system and a top view of the device.

**Figure 16:**
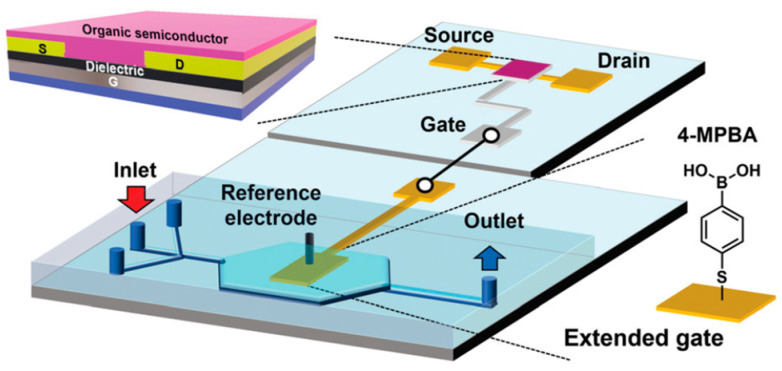
Schematic illustration of the microfluidic system with the extended-gate-type OFET sensor for H_2_O_2_ detection. The detection portion was functionalized with 4-MPBA. Reproduced from Ohshiro et al. [103] with permission from RSC Publishing.

In a work that followed, the development of a potentially portable microfluidic–Au extended-gate-type OFET sensor for reversible and real time continuous H_2_O_2_ monitoring, with a 0.71 pg/mL detection limit in ultra-pure water, was reported. This transistor structure (see Figure 16) protected its organic semiconductor from possible instability in water. The organic channel was also protected by a passivating layer of Cytop, enabling operation under ambient conditions [103].

The extended-gate electrode, incorporated within the PDMS microfluidic chip, was functionalized with 4-MPBA, which is oxidized by H_2_O_2_ to form a phenol that is hydrolyzed in water, and a boronate anion that affects *V_th_*. The H_2_O_2_-level-dependent *I_DS_* and *V_th_* in the transfer characteristics (*I_DS_* vs. *V_GS_* at a given *V_DS_* = −1 V) before and after adding different levels of H_2_O_2_ were measured. Using these data, a calibration curve, i.e., the difference in *V_th_* normalized to that in the absence of [H_2_O_2_], was generated.

As mentioned in the Background section, EGTs of organic semiconductors are divided into two main classes—EGOFETs and OECTs. The fabrication and biosensing operation of EGTs was overviewed by Dorfman et al. [104]. In particular, they discussed improvement in the sensing performance by integrating such printed transistors with microfluidics, which enabled multiplexing, sample preconcentration, and improved transport.

Spanu et al. [105] reviewed in vitro and in vivo applications of organic transistors interfaced to cells; EGOFETs and OECTs were among the structures reviewed. In particular, they reviewed and compared OECTs, two types of EGOFETs (solution-gated OFETs and solution-gated graphene transistors), and organic-charge-modulated FETs. As shown in the following examples, EGOFETs and OECTs that can operate in aqueous solutions are very promising organic bioelectronic tools for broad biosensing and biomedical applications, and their integration with microfluidics enables development of low-cost compact, improved devices for potentially portable POC testing, as well as for in vitro and in vivo studies.

#### 2.2.1. Examples of EGOFETs

The label-free detection of different analytes is possible with various integrated EGOFETs-microfluidic designs. As an example, Berto et al. [106] reported a single-gated EGOFET with surface-immobilized recognition peptide aptamers for the detection of the tumor necrosis factor alpha (TNFα), which is an inflammatory biomarker; the LOD was ~1 pM.

**Figure 17:**
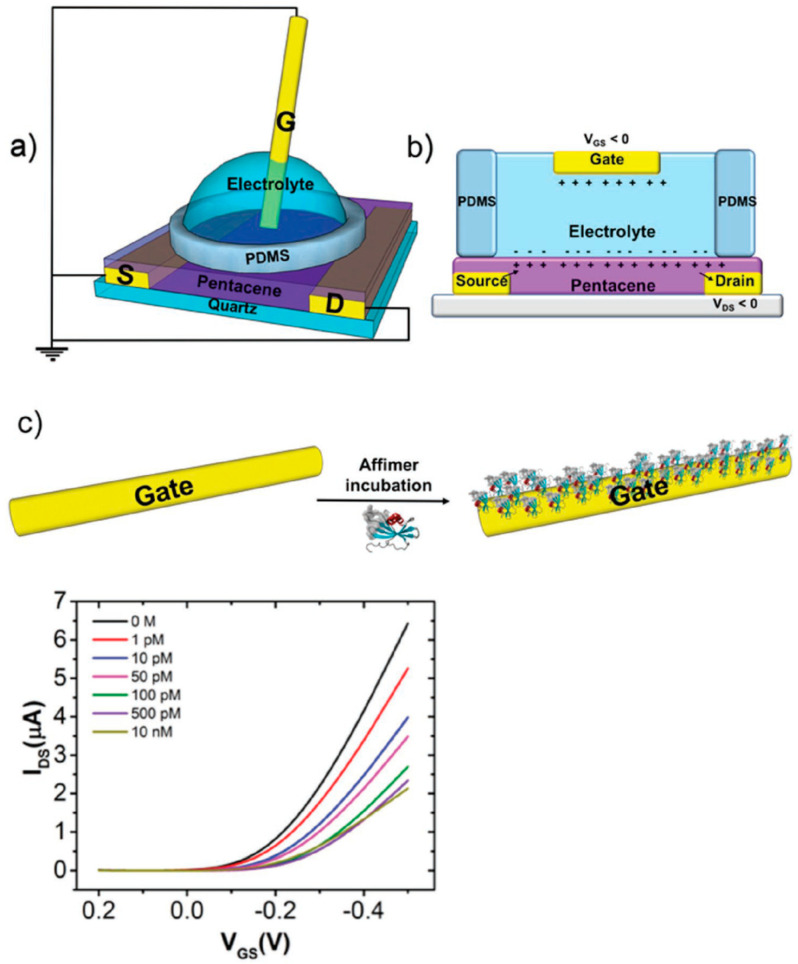
Top: (**a**) schematic drawing of the experimental setup with electrical connections and (**b**) schematic of the EGOFET cross-section. (**c**) Functionalization of the gate electrode: anti-TNFα peptide aptamers directly immobilized on the gold surface. Bottom: transfer characteristics of the EGOFET-based sensor resulting from exposure to different [TNFα] solutions in PBS (TNFα concentrations are reported in the legend). Reproduced from Berto et al. [106] with permission from Wiley–VCH GmbH.

Figure 17 shows the schematics of the experimental setup and the EGOFET cross-section, together with the functionalized Au-gate electrode. The device’s transfer characteristics (as mentioned, *I_DS_* vs. *V_GS_* at a given *V_DS_*) is often used to characterize sensor performance, with various concentrations of TNFα also shown. As shown, *I_DS_* decreased as [TNFα] increased.

A multigate device with an internal reference electrode was later developed by Pakula et al. [107]. The device successfully assessed the reproducibility, selectivity, and sensitivity of monitoring TNFα in a lab-on-a-chip. The analyte, contained in microliter volumes, was successfully monitored in minutes, with an LOD of 3 pM. As is typical, the device performance was evaluated via its transfer characteristic curves obtained by measuring *I_DS_* at various analyte levels, while sweeping *V_GS_* at a constant *V_DS_*. Reference measurements in the absence of the analyte were also conducted.

Ricci et al. developed a disposable coplanar EGOFET integrated with microfluidics for label-free, fast, and sensitive monitoring of the aggregation of *α*-synuclein, which is considered to be a marker of some neurological diseases [108]. A calibration curve was obtained from measurements of *I_DS_* vs. *V_GS_* at *V_DS_* = −0.1 V for different analyte concentrations; the LOD was 0.25 pM. The structure of the microfluidic chip, with a separately accommodated gate and organic semiconductor, enabled controllable contamination-free functionalization of the Au-gate electrode with monoclonal anti-(*α*-synuclein) antibodies.

Doumbia et al. [51] recently reported integrated EGOFET arrays-microfluidics on a flexible substrate for label-free continuous, in situ real-time detection (which has not often been explored for biomolecule binding) of DNA hybridization under a flowing electrolyte with <1 s temporal resolution.. The sensing was based on measuring a shift in the device’s *V_th_* that occurred due to a change in the gate electrode work function upon DNA hybridization.

OECTs, the structure of which is similar to that of EGOFETs, have also been used in a variety of biological and chemical applications, and extensively reported; some recent examples are provided below.

As mentioned, in EGOFETs, the semiconductor is ion-impermeable. Biasing the gate and the drain results in an electrical double layer at the electrolyte-semiconductor interface. In contrast, in OECTs, the channel’s volume is ion-permeable.

#### 2.2.2. Examples of OECTs

The biocompatible PEDOT:PSS, an ionic-electronic conducting polymer, usually serves as the channel. The device operation is based on the channel’s electrochemical doping/de-doping, with ions entering the active region following ~0.5 V gate biasing.

**Figure 18:**
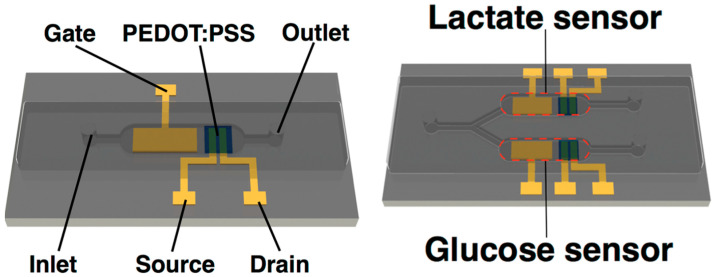
Left: schematic diagram of glucose sensor based on OECT integrated with a microfluidic channel. Right: schematic diagram of multi-analyte sensor. Reproduced from Ji et al. [109] with permission from Wiley–VCH GmbH.

Ji et al. [109] reported sensitive and fast glucose and lactate sensors, based on Pt nanoparticles and an OECT with the p-type PEDOT:PSS channel integrated with a PDMS microfluidic structure that enabled use of small volumes. The detection of glucose in saliva, as well as the simultaneous monitoring of both glucose and lactate in two separate channels on a single chip, were demonstrated; the LODs were 10^−7^ M and 10^−6^ M, respectively. Figure 18 shows schematic diagrams of the integrated sensor and the multianalyte structure. Additionally, using a smartphone and Bluetooth connection, a prototype of a portable glucose sensor for real-time sensing was fabricated, showing the potential of the approach for portable healthcare monitoring.

Koklu et al. [110] presented a glucose sensor based on the first integrated microfluidic-n-type OECT operating in the accumulation mode. The sensitivity and limit of detection of the integrated device, down to the nM range, improved, in comparison to a non-integrated device.

**Figure 19:**
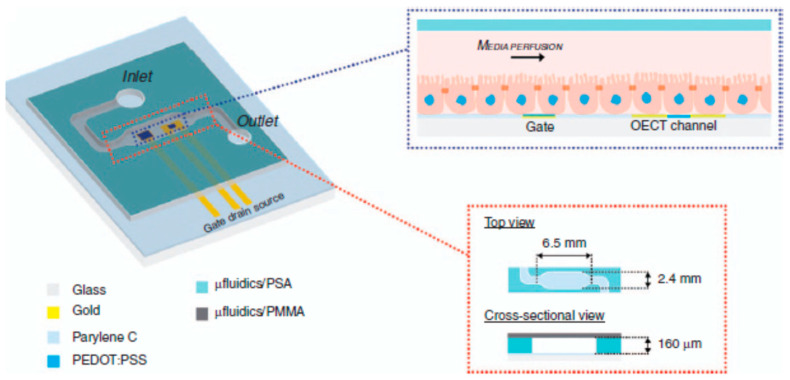
The integration of microfluidics with the OECT for combined optical and electronic monitoring. Left: graphical representation of the developed platform integrating the OECT with microfluidics. Top right, an illustration of the OECTs and the cell layer lining the bottom surface of the microfluidic channel. Bottom right, top and cross-sectional views of the microfluidic device. Reproduced from Curto et al. [53] with permission from Springer Nature.

The first sensor based on an OECT that was integrated with microfluidics for in vitro cell multiparameter monitoring was reported by Curto et al. [53]. This integration, in conjunction with different methods—e.g., optical imaging, impedance monitoring, and metabolite sensing—was presented. Figure 19 shows the schematics of the integrated platform and a cross-section of the device. This design, with the inclusion of in-line sensors in microfluidic structures, enabled real-time monitoring. It successfully used different monitoring techniques to evaluate cell morphology, differentiation, and integrity.

Another OCET example is that of a low-power, low-volume, broad-dynamic-range, and sensitive OECT-microfluidic immunosensor for label-free Alzheimer’s-disease monitoring via detection of Amyloid-β protein aggregates in human serum [111]. A nanoporous membrane functionalized with Congo red molecules that showed a selective, strong affinity to the aggregates was used. Upon aggregate binding, the OECT’s characteristics, e.g., its capacitance and *I_DS_*, changed. The authors evaluated the device performance using three different channel materials—the intrinsically-doped PEDOT:PSS, which operates in the depletion mode, and synthesized p- and n-type semiconducting polymers that operated in the accumulation mode, which resulted in lower power and higher sensitivity devices—with the n-type material, showing superior performance.

To achieve selectivity and high sensitivity in monitoring biomolecules, an OECT immunosensor for Thrombin (a protein involved in blood coagulation), with a gate electrode based on polyethylene film-multilayer graphene composite/gold nanoparticles/Thrombin-binding aptamers, was developed. Due to this gate-multilayer-electrode structure, the selective detection of Thrombin was enabled. A high sensitivity at a picomolar level (LOD < 5 pM) was possible, due to the large number of gold particles/aptamers at the electrode’s surface; the channel material was PEDOT:PSS [112].

## 3. Summary and Concluding Remarks

In this review, we presented examples that showed the promise of OE–LOC/microfluidics integration for biochemical sensing and biomedical applications. The advantageous small-size and highly sensitive OE devices included OLEDs, OPDs, and PPDs for optical monitoring and OFETs (notably OECTs and EGOFETs) where solution analytes and biological processes affect the electrical properties of the organic transistor.

In optical sensing, we presented examples of sensitively and selectively monitoring analytes such as ethanol, glucose, and lactate, as well as agricultural and other chemical toxins. OLEDs, OPDs, and PPDs are easily and inexpensively fabricated at ambient temperature on glass and plastic and are, therefore, compatible with microfluidic structures. Indeed, sensitive OLED/OPD-microfluidic monitors, including multiplexed optical OLED/OPD sensors on flexible PET, were demonstrated, showing promise for medical applications as inexpensive and reliable wearable and portable POC PL monitors.

In non-optical sensing, we presented the promise of integrated OFET/microfluidics-based bioelectronic tools, including extended-gate OFETs, EGOFETs, and OECTs. The examples showed the advances achieved using integrated devices. Sensitive, real-time glucose and continuous H_2_O_2_ monitoring were demonstrated using extended-gate OFETs. Integrated EGOFETs and OECTs showed improved performance, potentially enabling low-cost portable POC testing, as well as improved devices for in vitro and in vivo studies. The promise of integrated EGOFET-microfluidic tools for monitoring label-free disease markers and DNA hybridization, including lab-on-a-chip, was also shown.

Promising examples of integrated OECT-microfluidics were also presented for, e.g., fast glucose and lactate sensing, in vitro cell multiparameter monitoring, and immunosensors such as sensitive low-power, low-volume immunosensors for label-free Alzheimer’s disease monitoring and Thrombin detection.

Research and development of OE bioelectronic tools integrated with microfluidic structures is ongoing, due to its great potential in improving efficient and broad sensing and medical-monitoring applications. Versatile OE devices integrated with microfluidics have improved sensitivity, selectivity, and response times. The continued development of OE devices for other general applications, such as solar cells and SSL, promise improved monitors with long-term stability, and the development of new materials in, e.g., OFETs should further improve their performance with reproducible and selective applications, including applications in wearable devices.

## Data Availability

No new data were created or analyzed in this study. Data sharing is not applicable to this article.
